# Impact of the COVID-19 Pandemic on Kidney Diseases Requiring Renal Biopsy: A Single Center Observational Study

**DOI:** 10.3389/fphys.2021.649336

**Published:** 2021-07-08

**Authors:** Samy Hakroush, Désirée Tampe, Peter Korsten, Björn Tampe

**Affiliations:** ^1^Institute of Pathology, University Medical Center Göttingen, Göttingen, Germany; ^2^Department of Nephrology and Rheumatology, University Medical Center Göttingen, Göttingen, Germany

**Keywords:** COVID-19 pandemic, COVID-19 lockdown, kidney disease, renal biopsy, hypertensive nephropathy, hemoglobinuria, proteinuria

## Abstract

**Background:**

The coronavirus disease-2019 (COVID-19) pandemic impacted healthcare services for kidney disease patients. Lockdown and social distancing were mandated worldwide, resulting in closure of medical services. The diagnosis of various kidney diseases may have been delayed during the COVID-19 pandemic because non-urgent tests and visits were postponed due to closure of medical services during the lockdown.

**Methods:**

We here report the impact of the COVID-19 pandemic on a total number of 209 native kidney diseases requiring renal biopsy for diagnosis in a retrospective observational study from a tertiary hospital in Germany.

**Results:**

The lockdown period in March and April 2020 primarily affected patients admitted to the normal medical ward with a compensatory increased rate of renal biopsies in the postlockdown phase. In addition, there was a shift toward more patients admitted with hemoglobinuria during the COVID-19 pandemic. This phenomenon of an increased number of patients with hemoglobinuria during the COVID-19 pandemic was specifically observed in a subgroup with hypertensive nephropathy requiring renal biopsy and associated with increased proteinuria, not attributed to the COVID-19 lockdown period itself.

**Conclusion:**

To our knowledge, this is the first report of identifying a subpopulation susceptible to closure of medical services during the COVID-19 pandemic and diagnostic delay of specific kidney diseases. Therefore, the COVID-19 pandemic should be regarded as a risk factor especially in patients with diseases other than COVID-19 primarily admitted to the normal medical ward.

## Introduction

The coronavirus disease-2019 (COVID-19) pandemic impacted healthcare services for kidney disease patients. Lockdown and social distancing were mandated worldwide, resulting in closure of medical services. The diagnosis of various kidney diseases may have been delayed during the COVID-19 pandemic because non-urgent tests and visits were postponed due to closure of medical services during the lockdown (Prasad et al., 2020). Based on previous reports, this affects especially kidney diseases requiring renal biopsy and histological analysis for diagnosis (Chuah et al., 2020; Gauckler et al., 2020; Giollo et al., 2020; Hussein et al., 2020; Hakroush and Tampe, 2021). We have previously reported that during the lockdown period in March and April 2020, an incidence-shift with a COVID-19 gap of no diagnosed antineutrophil cytoplasm antibodies (ANCA)-associated vasculitis (AAV) and ANCA glomerulonephritis (GN) based on renal biopsy was followed by a postlockdown phase in subsequent months with a compensatory increased incidence rate (Hakroush and Tampe, 2021). This has been attributed to a decreased number of renal biopsies during the lockdown period and a compensatory increased number in the postlockdown phase (Hakroush and Tampe, 2021). We here expanded our analysis to evaluate the effect of the COVID-19 pandemic on kidney diseases requiring renal biopsy. Furthermore, we aimed to identify effects of the COVID-19 pandemic on clinical outcomes in patients with kidney diseases. With multiple vaccines currently undergoing human trials to combat this pandemic, there is an urgent need for a clear sense for patient populations most susceptible to shutdown of medical services. We here report the impact of the COVID-19 pandemic on native kidney diseases requiring renal biopsy for diagnosis in a retrospective observational study from a tertiary hospital in Germany.

## Materials and Methods

### Study Population

**FIGURE 1:**
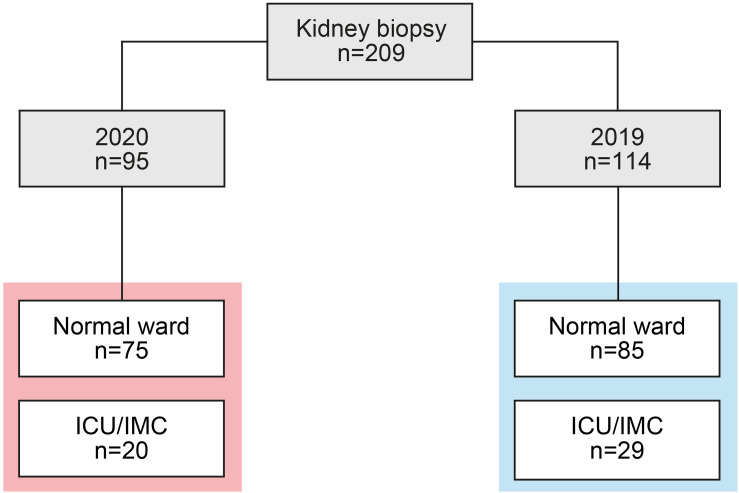
Total cohort of renal biopsies. STROBE flow chart of patient disposition, RRT was performed intermittently in all cases. STROBE, Strengthening the Reporting of Observational Studies in Epidemiology.

A total number of 209 renal biopsies performed on native kidneys of patients hospitalized at the University Medical Center Göttingen in 2019 and 2020 were included. The indication for kidney biopsy included clinical or serologic evidence of a systemic disease, acute nephritic syndrome, unexplained deterioration of kidney function, nephrotic syndrome, non-nephrotic proteinuria of more than 1000 mg per day and glomerular hematuria. In contrast, we do not routinely perform renal biopsies in critically ill patients with ischemic kidney injury. In addition, all patients admitted during the COVID-19 pandemic were tested negative for severe acute respiratory syndrome coronavirus type 2 (SARS-CoV-2). While no formal approval was required for the use of routine clinical data, a favorable ethical opinion was granted by the local Ethics committee (no. 28/9/17). A detailed Strengthening the Reporting of Observational Studies in Epidemiology (STROBE) flow chart of patient disposition is shown in Figure 1.

### Statistical Methods

Variables were tested for normal distribution using Shapiro-Wilk test. Non-normally distributed continuous variables are expressed as median and interquartile range (IQR), categorical variables are presented as frequencies and percentages from the total number with parameters available. Statistical comparisons were not formally powered or prespecified. For group comparisons, the Mann-Whitney *U*-test was used to determine differences in medians. Non-parametric between-group-comparisons were performed with Pearson’s Chi-square test. Data analyses were performed with GraphPad Prism (version 8.4.0 for MacOS, GraphPad Software, San Diego, CA, United States).

## Results

### Comparison of Renal Biopsies Performed in 2019 and 2020 During the COVID-19 Pandemic

**FIGURE 2:**
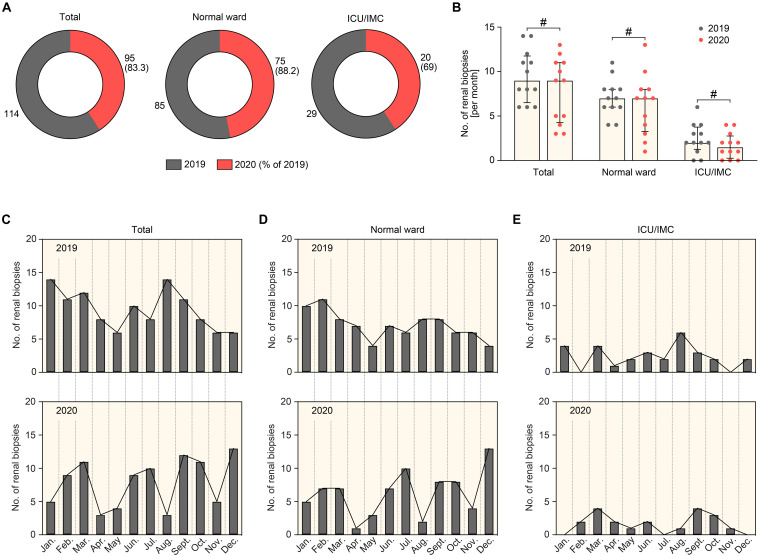
Comparison of renal biopsies performed in 2019 and 2020 during the COVID-19 pandemic. **(A)** Total number of renal biopsies in 2019 and 2020 in total patients, patients admitted to the normal or ICU/IMC medical ward. **(B)** The scatter dot plots represent medians and IQR with individual data points summarizing the number of renal biopsies per month in 2019 compared to 2020, the Mann-Whitney *U*-test was used to determine differences in medians and # indicates no statistic significance. **(C–E)** Absolute number of renal biopsies per month in 2019 and 2020 in total patients, patients admitted to the normal or ICU/IMC medical ward. Apr., April; Aug., August; Dec., December; Feb., February; ICU, intensive care unit; IMC, intermediate care unit; Jan., January; Jul., July; Jun., June; Mar., March; No., number; Nov., November; Oct., October; Sept., September.

A total number of 209 renal biopsies were performed in 2019 and 2020 and included in our study (Figure 1). During the COVID-19 pandemic, we observed a non-significant reduction of total renal biopsies performed per month, including the normal medical ward and patients primarily admitted to the intermediate care (IMC) and intensive care unit (ICU, Figures 2A,B). We next analyzed the effect of the lockdown period on renal biopsies performed in 2020 compared to 2019. In line with previous reports, an incidence-shift with a COVID-19 gap during the lockdown period in March and April 2020 was followed by a postlockdown phase in subsequent months with a compensatory increased rate of renal biopsies (Figure 2C; Chuah et al., 2020; Gauckler et al., 2020; Giollo et al., 2020; Hussein et al., 2020; Hakroush and Tampe, 2021). The COVID-19 gap preferentially affected patients admitted to the normal medical ward, in line with closure of medical services for regular care (Figure 2D). In contrast, no such COVID-19 gap was observed in critically ill patients requiring ICU/IMC supportive care (Figure 2E). In summary, there was a non-significant reduction of renal biopsies during the COVID-19 pandemic affecting both, patients admitted to the normal medical ward and ICU/IMC. Furthermore, the lockdown period in March and April 2020 primarily affected patients admitted to the normal medical ward with a compensatory increased rate of renal biopsies in the postlockdown phase. In contrast, no such COVID-19 gap was observed in patients admitted to the ICU/IMC medical wards, in line with shutdown of services involved in regular medical care.

### Comparison of Clinical and Laboratory Markers in Patients Requiring Renal Biopsy in 2019 and 2020 During the COVID-19 Pandemic and Lockdown Period in March and April 2020

**FIGURE 3:**
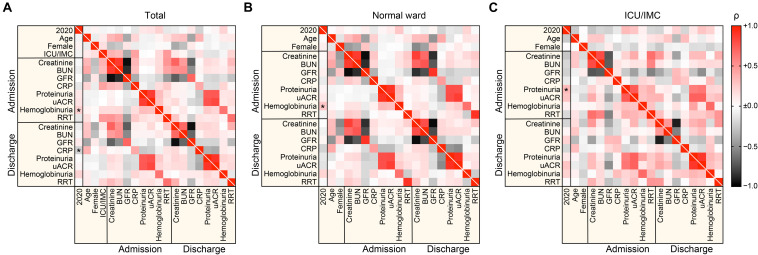
Comparison of clinical and laboratory markers in patients requiring renal biopsy in 2019 and 2020 during the COVID-19 pandemic. **(A–C)** Association between renal biopsies performed in 2020 during the COVID-19 pandemic, clinical and laboratory findings at admission and discharge are shown by heatmap reflecting mean values of Spearman’s ρ, asterisks indicate *p* < *0.05*. BUN, blood urea nitrogen; CRP, C-reactive protein; GFR, glomerular filtration rate (CKD-EPI); ICU, intensive care unit; IMC, intermediate care unit; RRT, renal replacement therapy; uACR, urinary albumin:creatinine ratio.

We next assessed diagnoses of kidney diseases in renal biopsies during the COVID-19 pandemic in comparison to 2019. There was no significant change in diagnoses of kidney diseases based on renal biopsies (Table 1), indicating that the COVID-19 pandemic does not affect the overall distribution of kidney diseases. Based on these observations that the COVID-19 pandemic did not affect the distribution of kidney diseases in patients admitted to our center, we next compared clinical and laboratory measurements of patients with kidney diseases requiring renal biopsy comparing the COVID-19 pandemic with 2019. Interestingly, we identified that significantly more patients were admitted with hemoglobinuria during the COVID-19 pandemic as compared to 2019 (Figure 3A and Table 2). In line with our previous observation that the COVID-19 pandemic resulted in a reduced number of renal biopsies performed during the COVID-19 gap, the increase in hemoglobinuria was attributed to patients admitted to the normal medical ward (Figure 3B and Table 2). In addition, patients primarily admitted to the ICU/IMC medical ward during the COVID-19 pandemic displayed significantly more proteinuria as compared to 2019 (Figure 3C and Table 2). While an increase in patients with hemoglobinuria admitted to the normal ward and more proteinuria specifically in patients admitted to the ICU/IMC medical ward were observed during the COVID-19 pandemic, laboratory markers at discharge during the COVID-19 pandemic were comparable to 2019 (Figures 3A–C and Table 2), suggesting that short-term clinical outcomes were equally distributed. To gain insights into distinct characteristics during the COVID-19 lockdown period in March and April 2020, we next directly compared clinical and laboratory measurements of patients with kidney diseases requiring renal biopsy comparing March/April 2020 with March/April 2019. In line with our aforementioned findings, an increase in patients with hemoglobinuria was observed during the COVID-19 lockdown period (Table 3). In summary, we here show that the COVID-19 pandemic predominantly affected patients with kidney diseases requiring renal biopsy admitted to the normal medical ward. Furthermore, we observed more hemoglobinuria in patients primarily admitted to the normal ward and more proteinuria specifically in patients admitted to the ICU/IMC medical ward.

**TABLE 1:** Diagnoses of kidney diseases based on renal biopsies comparing 2020 with 2019.

Diagnosis	2020	2019	*P* value
Total – no.	95	114	
ANCA GN – no. (%)	15 (15.8)	17 (14.9)	
Acute interstitial nephritis – no. (%)	9 (9.5)	18 (15.8)	
IgA nephropathy – no. (%)	8 (8.4)	10 (8.8)	
Diabetic nephropathy – no. (%)	7 (7.4)	13 (11.4)	
FSGS – no. (%)	7 (7.4)	15 (13.2)	
Hypertensive nephropathy – no. (%)	7 (7.4)	9 (7.9)	
Lupus nephritis – no. (%)	7 (7.4)	9 (7.9)	
IgA vasculitis – no. (%)	3 (3.2)	2 (1.8)	
Membranous GN – no. (%)	3 (3.2)	4 (3.5)	
Postinfectious GN – no. (%)	3 (3.2)	1 (0,9)	
Minimal change disease – no. (%)	3 (3.2)	1 (0,9)	
Thrombotic microangiopathy – no. (%)	2 (2.1)	2 (1.8)	
Amyloidosis – no. (%)	2 (2.1)	1 (0,9)	
Monoclonal immune deposition disease – no. (%)	2 (2.1)	2 (1.8)	
Others/not representative – no. (%)	17 (17.9)	10 (8.8)	*0.6532*

*Non-parametric between-group-comparisons were performed with Pearson’s Chi-square test. ANCA, anti-neutrophil cytoplasmic antibodies; BUN, blood urea nitrogen; CRP, C-reactive protein; FSGS, focal segmental glomerulosclerosis; GN, glomerulonephritis; IgA, immunoglobulin A; IQR, interquartile range; No., number.*

**TABLE 2:** Renal biopsies and characteristics of patients comparing 2020 with 2019.

	2020	2019	*P* value
**Total**			
No. of renal biopsies	95	114	
Renal biopsies per month (IQR) – no.	9 (4.25–11)	9 (6.5–11.8)	*0.3091*
Age (IQR) – years	58 (47–69)	60 (47.5–72.3)	*0.4041*
Female sex – no. (%)	46 (48.4)	49 (43)	*0.4317*
Creatinine at admission (IQR) – mg/dL	1.83 (1.15–3.07)	1.99 (0.97–3.99)	*0.6766*
eGFR at admission (IQR) – mL/min	42.6 (20–59.4)	32.7 (20–60)	*0.9684*
BUN at admission (IQR) – mg/dL	27 (18–48)	31.5 (18.3–57.8)	*0.2778*
Proteinuria at admission (IQR) – mg/L	754 (337–2638)	633 (181–1865)	*0.1070*
uACR at admission (IQR) – mg/g	694 (129–2907)	475 (47.4–1782)	*0.0585*
Hemoglobinuria at admission – no. (%)	71 (77.2)	64 (58.2)	***0.0043***
Creatinine at discharge (IQR) – mg/dL	1.93 (1.32–2.69)	2.18 (1.27–3.23)	*0.3290*
eGFR at discharge (IQR) – mL/min	33.7 (21.8–53.3)	29.6 (20–51.3)	*0.3210*
BUN at discharge (IQR) – mg/dL	36 (25–53)	37 (21–59.5)	*0.7262*
Proteinuria at discharge (IQR) – mg/L	554 (165–1989)	739 (153–2121)	*0.9400*
uACR at discharge (IQR) – mg/g	605 (77.2–2524)	893 (30.3–2244)	*0.7073*
**Normal medical ward**			
No. of renal biopsies	75	85	
Creatinine at admission (IQR) – mg/dL	1.68 (1.03–2.81)	1.68 (0.89–2.67)	*0.7930*
eGFR at admission (IQR) – mL/min	53.4 (29.7–60)	40.3 (22.3–60)	*0.4012*
BUN at admission (IQR) – mg/dL	23 (16–41)	28 (16.3–47)	*0.6071*
Proteinuria at admission (IQR) – mg/L	499 (317–2805)	699 (238–2238)	*0.5937*
uACR at admission (IQR) – mg/g	573 (106–2505)	491 (40.6–2159)	*0.2660*
Hemoglobinuria at admission – no. (%)	54 (75)	41 (50.6)	***0.0019***
Creatinine at discharge (IQR) – mg/dL	1.97 (1.33–2.69)	2.01 (1.1–2.72)	*0.9606*
eGFR at discharge (IQR) – mL/min	36.3 (21.9–53.3)	32 (20.5–52.9)	*0.7231*
BUN at discharge (IQR) – mg/dL	33 (23–53)	36 (19.5–61)	*0.5101*
Proteinuria at discharge (IQR) – mg/L	543 (179–1918)	1195 (259–3361)	*0.3915*
uACR at discharge (IQR) – mg/g	556 (102–2157)	1079 (180–4599)	*0.3595*
**ICU/IMC medical ward**			
No. of renal biopsies	20	29	
Creatinine at admission (IQR) – mg/dL	3.36 (1.48–4.16)	5.25 (1.97–8.67)	*0.0645*
eGFR at admission (IQR) – mL/min	20 (18.8–26.9)	20 (20–28.6)	*0.1947*
BUN at admission (IQR) – mg/dL	37 (25.3–69.8)	55.5 (31.3–85.8)	*0.1985*
Proteinuria at admission (IQR) – mg/L	1288 (770–2549)	455 (138–1573)	***0.0236***
uACR at admission (IQR) – mg/g	1143 (267–3507)	380 (53.4–1154)	*0.0567*
Hemoglobinuria at admission – no. (%)	17 (85)	23 79.3)	*0.6132*
Creatinine at discharge (IQR) – mg/dL	1.79 (1.32–3.02)	2.41 (1.36–4.77)	*0.1773*
eGFR at discharge (IQR) – mL/min	32 (20.6–53.3)	20 (20–44.6)	*0.2007*
BUN at discharge (IQR) – mg/dL	40 (30.3–55.5)	37.5 (22.3–50)	*0.7363*
Proteinuria at discharge (IQR) – mg/L	987 (152–2134)	428 (141–1302)	*0.5337*
uACR at discharge (IQR) – mg/g	1249 (64–3476)	395 (24.3–1461)	*0.1599*

*For group comparisons, the Mann-Whitney *U*-test was used to determine differences in medians. Non-parametric between-group-comparisons were performed with Pearson’s Chi-square test. Median values are shown, bold indicates statistically significant values at group level. BUN, blood urea nitrogen; CRP, C-reactive protein; ICU, intensive care unit; IMC, intermediate care unit; IQR, interquartile range; No., number; uACR, albumin:creatinine ratio.*

**TABLE 3:** Renal biopsies and characteristics of patients comparing the COVID-19 lockdown period 2020 with the same period in 2019.

Parameter	March/April 2020	March/April 2019	*P* value
No. of renal biopsies	14	20	
Age (IQR) – years	52.5 (35–69)	58.5 (34.5–67.8)	*0.6101*
Female sex – no. (%)	3 (21.4)	7 (35)	*0.3927*
Creatinine at admission (IQR) – mg/dL	1.74 (1.32–4.23)	1.55 (0.873–4.27)	*0.4160*
eGFR at admission (IQR) – mL/min	42.6 (20–55.1)	41.2 (20–60)	*0.3522*
BUN at admission (IQR) – mg/dL	33 (20.5–52.5)	28 (15–63)	*0.7890*
Proteinuria at admission (IQR) – mg/L	1135 (654–2439)	966 (242–2195)	*0.4643*
uACR at admission (IQR) – mg/g	743 (170–2079)	590 (43.8–1573)	*0.4415*
Hemoglobinuria at admission – no. (%)	13 (92.3)	10 (50)	***0.0086***
Creatinine at discharge (IQR) – mg/dL	1.81 (1.32–3.01)	1.76 (1.1–2.42)	*0.4486*
eGFR at discharge (IQR) – mL/min	35.3 (20.6–53.3)	39.7 (20–60)	*0.7092*
BUN at discharge (IQR) – mg/dL	43 (25–52)	27 (17–50)	*0.4878*
Proteinuria at discharge (IQR) – mg/L	1918 (605–2134)	1070 (605–2129)	*0.4908*
uACR at discharge (IQR) – mg/g	1474 (217–2182)	642 (108–1517)	*0.4206*

*For group comparisons, the Mann-Whitney *U*-test was used to determine differences in medians. Non-parametric between-group-comparisons were performed with Pearson’s Chi-square test. Median values are shown, bold indicates statistically significant values at group level. BUN, blood urea nitrogen; CRP, C-reactive protein; ICU, intensive care unit; IMC, intermediate care unit; IQR, interquartile range; No., number; uACR, albumin:creatinine ratio.*

### Comparison of Clinical and Laboratory Markers Among Distinct Kidney Diseases in 2019 and 2020 During the COVID-19 Pandemic

Based on our observation that more patients were admitted with hemoglobinuria and proteinuria as compared to 2019, we next aimed to gain insights into distinct kidney diseases affected by increased hemoglobinuria during the COVID-19 pandemic. Interestingly, we identified that the increase in hemoglobinuria was specifically attributed to patients with hypertensive nephropathy during the COVID-19 pandemic because no such phenomenon was observed in other kidney diseases as compared to 2019 (Table 4). This is in line with our previous observation that during the lockdown period in March and April 2020, an incidence-shift with a COVID-19 gap of non-diagnosed ANCA GN based on renal biopsy was followed by a postlockdown phase in subsequent months (Hakroush and Tampe, 2021). Interestingly, higher incidence of hemoglobinuria among patients admitted with hypertensive nephropathy was associated with increased proteinuria (Table 4). This was not attributed to the COVID-19 lockdown itself, since no hypertensive nephropathy was diagnosed in the period between March and April 2020 (Table 5). In contrast, laboratory markers at discharge during the COVID-19 pandemic were comparable to 2019 among all kidney diseases (Table 6), implicating that short-term clinical outcomes were equally distributed. In summary, we identified that the shift toward more patients admitted with hemoglobinuria and proteinuria during the COVID-19 pandemic was specifically observed in a subgroup with hypertensive nephropathy requiring renal biopsy and not attributed to the COVID-19 lockdown period itself.

**TABLE 4:** Laboratory findings at admission in kidney diseases based on renal biopsies comparing 2020 with 2019.

	2020	2019	*P* value
**ANCA GN**			
Median creatinine (IQR) – mg/dL	3.26 (1.2–3.96)	2.28 (1.12–4.8)	*0.9111*
Median BUN (IQR) – mg/dL	33 (19–39)	43 (24.8–75.8)	*0.1219*
Median proteinuria (IQR) – mg/L	812 (476–3584)	1334 (549–1898)	*0.7375*
Median uACR (IQR) – mg/g	1222 (376–4925)	703 (217–1047)	*0.1645*
Median hemoglobinuria– no. (%)	6 (66.7)	17 (100)	***0.0114***
**Acute interstitial nephritis**			
Median creatinine (IQR) – mg/dL	1.07 (0.975–2)	2.78 (2.1–8.55)	***0.0091***
Median BUN (IQR) – mg/dL	20 (16.5–46)	48 (24.3–80.8)	***0.0343***
Median proteinuria (IQR) – mg/L	459 (353–2357)	207 (129–859)	***0.0268***
Median uACR (IQR) – mg/g	1313 (267–2099)	53.3 (30–654)	***0.0294***
Median hemoglobinuria– no. (%)	7 (87.5)	5 (50)	*0.0935*
**IgA nephropathy**			
Median creatinine (IQR) – mg/dL	1.5 (0.935–3.49)	2.18 (1.28–4.87)	*0.5726*
Median BUN (IQR) – mg/dL	26.5 (20.5–73.3)	35.5 (17.8–56)	*0.9844*
Median proteinuria (IQR) – mg/L	563 (256–1380)	1252 (792–2168)	*0.2743*
Median uACR (IQR) – mg/g	399 (105–1867)	933 (298–1755)	*0.5726*
Median hemoglobinuria– no. (%)	6 (75)	9 (90)	*0.3961*
**Diabetic nephropathy**			
Median creatinine (IQR) – mg/dL	1.29 (0.76–1.52)	2.5 (1.98–4.24)	***0.0047***
Median BUN (IQR) – mg/dL	17 (13–19)	46 (31.5–77.5)	***0.0032***
Median proteinuria (IQR) – mg/L	2536 (214–5755)	623 (165–2861)	*0.6426*
Median uACR (IQR) – mg/g	2167 (135–3371)	491 (31.9–3737)	*0.6426*
Median hemoglobinuria– no. (%)	4 (57.1)	6 (46.2)	*0.6392*
**FSGS**			
Median creatinine (IQR) – mg/dL	1.68 (1.24–2.47)	1.59 (0.758–2.96)	*0.6359*
Median BUN (IQR) – mg/dL	32 (20–62.5)	28 (16.5–53.5)	*0.5519*
Median proteinuria (IQR) – mg/L	368 (336–1104)	750 (159–2409)	*0.5846*
Median uACR (IQR) – mg/g	226 (34.4–694)	1175 (309–4218)	*0.1718*
Median hemoglobinuria– no. (%)	4 (66.7)	7 (50)	*0.4924*
**Hypertensive nephropathy**			
Median creatinine (IQR) – mg/dL	2.69 (1.32–3.36)	1.49 (1.2–2.2)	*0.2105*
Median BUN (IQR) – mg/dL	48 (25–49)	31 (19.5–64.5)	*0.4066*
Median proteinuria (IQR) – mg/L	860 (258–3000)	132 (50.6–476)	***0.0093***
Median uACR (IQR) – mg/g	300 (112–1948)	29 (13.5–518)	*0.0813*
Median hemoglobinuria– no. (%)	6 (85.7)	1 (12.5)	***0.0046***
**Lupus nephritis**			
Median creatinine (IQR) – mg/dL	1.91 (0.73–2.12)	0.85 (0.655–1.37)	*0.1738*
Median BUN (IQR) – mg/dL	22 (12–31)	13 (11.5–18)	*0.1620*
Median proteinuria (IQR) – mg/L	372 (169–765)	1417 (67.8–2584)	*0.5350*
Median uACR (IQR) – mg/g	106 (68.4–2505)	1337 (30–3337)	*0.7104*
Median hemoglobinuria– no. (%)	5 (71.4)	4 (50)	*0.3980*

*For group comparisons, the Mann-Whitney *U*-test was used to determine differences in medians. Non-parametric between-group-comparisons were performed with Pearson’s Chi-square test. Bold indicates statistically significant values at group level. ANCA, anti-neutrophil cytoplasmic antibodies; BUN, blood urea nitrogen; CRP, C-reactive protein; FSGS, focal segmental glomerulosclerosis; GN, glomerulonephritis; IgA, immunoglobulin A; IQR, interquartile range; No., number; uACR, albumin:creatinine ratio.*

**TABLE 5:** Diagnoses of kidney diseases based on renal biopsies comparing the COVID-19 lockdown period 2020 with the same period in 2019.

Diagnosis	March/April 2020	March/April 2019	*P* value
Total – no.	14	20	
ANCA GN – no. (%)	3	4	
Acute interstitial nephritis – no. (%)	1	2	
IgA nephropathy – no. (%)	3	1	
Diabetic nephropathy – no. (%)	3	2	
FSGS – no. (%)	0	1	
Lupus nephritis – no. (%)	0	3	
IgA vasculitis – no. (%)	1	0	
Membranous GN – no. (%)	0	1	
Postinfectious GN – no. (%)	1	0	
Thrombotic microangiopathy – no. (%)	1	1	
Amyloidosis – no. (%)	0	1	
Monoclonal immune deposition disease – no. (%)	0	1	
Others/not representative – no. (%)	1	3	*0.5325*

*Non-parametric between-group-comparisons were performed with Pearson’s Chi-square test. ANCA, anti-neutrophil cytoplasmic antibodies; BUN, blood urea nitrogen; CRP, C-reactive protein; FSGS, focal segmental glomerulosclerosis; GN, glomerulonephritis; IgA, immunoglobulin A; IQR, interquartile range; No., number.*

**TABLE 6:** Laboratory findings at discharge in kidney diseases based on renal biopsies comparing 2020 with 2019.

	2020	2019	*P* value
**ANCA GN**			
Median creatinine (IQR) – mg/dL	2.59 (1.1–3.49)	2.93 (1.53–4.04)	*0.7371*
Median BUN (IQR) – mg/dL	52 (24–63)	53 (21–85)	*0.6569*
**Acute interstital nephritis**			
Median creatinine (IQR) – mg/dL	2.25 (1.86–5.34)	2.26 (1.38–3.03)	*0.6867*
Median BUN (IQR) – mg/dL	53 (34.5–70)	35 (21.5–69)	*0.3990*
**IgA nephropathy**			
Median creatinine (IQR) – mg/dL	1.32 (1.08–1.77)	2.37 (1.5–5.35)	*0.1061*
Median BUN (IQR) – mg/dL	31 (16–45)	42 (28–51.5)	*0.3220*
**FSGS**			
Median creatinine (IQR) – mg/dL	1.65 (1.34–2.74)	1.59 (1.17–2.76)	*0.9497*
Median BUN (IQR) – mg/dL	41 (19.3 (57.5)	32 (18–41)	*0.6485*
**Hypertensive nephropathy**			
Median creatinine (IQR) – mg/dL	1.89 (1.41–3.2)	1.63 (1.09–2.03)	*0.4206*
Median BUN (IQR) – mg/dL	43 (31–68)	38 (21–84)	*0.8413*
**Lupus nephritis**			
Median creatinine (IQR) – mg/dL	2.06 (0.67–2.32)	0.87 (0.81–4.22)	*0.6167*
Median BUN (IQR) – mg/dL	32 (13–56)	12 (11–75)	*0.5583*

*For group comparisons, the Mann-Whitney *U*-test was used to determine differences in medians. Non-parametric between-group-comparisons were performed with Pearson’s Chi-square test. ANCA, anti-neutrophil cytoplasmic antibodies; BUN, blood urea nitrogen; FSGS, focal segmental glomerulosclerosis; GN, glomerulonephritis; IgA, immunoglobulin A; IQR, interquartile range.*

## Discussion

Prompt diagnoses of kidney diseases requiring kidney biopsy and affected by the COVID-19 pandemic are crucial in the disease management. Although definite conclusion on long-term clinical outcomes cannot yet be drawn, our observations indicate that the COVID-19 pandemic resulted only in a minor reduction of renal biopsies in patients admitted to our tertiary center. While the lockdown period with closure of medical services was associated with an overall reduction of renal biopsies mainly attributed to patients admitted to the normal medical ward, there was a compensatory increased number of renal biopsies in this patient population during the postlockdown phase. In contrast, the number of renal biopsies in patients requiring IMC/ICU supportive care remained unaffected during the COVID-19 pandemic. This is in line with our previous report that during the lockdown period in March and April 2020, an incidence-shift with a COVID-19 gap of non-diagnosed AAV based on renal biopsy was followed by a postlockdown phase in subsequent months with a compensatory increased incidence rate (Hakroush and Tampe, 2021). According to the literature, various kidney diseases are preceded by a prodromal phase characterized by constitutional symptoms (Monti et al., 2020). Diagnostic delay at this stage is common and has been demonstrated to be associated with mortality and end-stage renal disease (Koldingsnes and Nossent, 2002; Houben et al., 2017). During the lockdown period, diagnosis of kidney diseases can be delayed because non-urgent tests and visits might have been postponed due to closure of medical services (Prasad et al., 2020). In addition, containment measures and fear may have contributed to downplay constitutional symptoms and underestimate their need for medical attention, leading to worse health outcomes (Lau et al., 2020). There has been shown a clear link between chronic diseases and risk of COVID-19 as pre-existing diseases increase susceptibility for severe COVID-19 course and outcome (Hanlon et al., 2018; Pati et al., 2020; Sinclair and Abdelhafiz, 2020). Our findings underscore that the COVID-19 pandemic has also a significant impact on patients with chronic diseases other than COVID-19, including kidney diseases, as previously reported (Saqib et al., 2020). This is especially important in patients requiring renal biopsy for a definite diagnosis, as focused on in this study performed in a tertiary center. Although definite conclusions on clinical outcomes cannot yet be drawn, our observations indicate no detrimental effects of COVID-19 on short-term clinical outcomes of patients with various kidney diseases. Nonetheless, prompt diagnoses and referral to tertiary centers especially affected by the ongoing global COVID-19 pandemic are crucial in the disease management. We here identified patients with hypertensive nephropathy requiring renal biopsy for definite diagnosis at risk for increased hemoglobinuria and proteinuria during the COVID-19 pandemic. Interestingly, the increased number of patients with hemoglobinuria and proteinuria in the subgroup with hypertensive nephropathy was not attributed to the COVID-19 lockdown period itself, implicating a general effect during the COVID-19 pandemic. Although definite conclusion on clinical outcomes cannot be yet drawn, this observation could be of relevance in these patient subpopulations. It has previously been shown that hemoglobinuria and proteinuria are associated with a higher risk of progressive kidney disease and death (Apperloo et al., 1994; Orlandi et al., 2018). With regard to patients with biopsy-proven hypertensive nephropathy, hemoglobinuria has been reported in 30–55% of patients (Harvey et al., 1992; Nonaka et al., 2013; Wang et al., 2013). This is especially relevant because hemoglobinuria has also been associated with progressive kidney disease and dialysis (Shantsila et al., 2012). In addition, proteinuria is the most accurate predictor of renal outcome and an independent predictor of mortality in patients with kidney diseases (Ruggenenti et al., 2000; de Zeeuw et al., 2004; Tonelli et al., 2006). Since the COVID-19 pandemic is ongoing and multiple vaccines currently undergoing human trials to combat this pandemic, there is an urgent need for a clear sense of patient populations most susceptible to closure of medical services.

While strict preventive measures are necessary to protect public health, they may also have an important impact on dietary and lifestyle behaviors. Staying and working at home can impact diet and food choice, affecting lifestyle behavior including physical activity (Guthold et al., 2018; Ammar et al., 2020). Beside a negative effect on physical activity during the COVID-19 pandemic, also dietary behavior changes with regard to increased food and alcohol intake were observed (Ammar et al., 2020; Gornicka et al., 2020). These dietary and lifestyle behaviors may contribute to metabolic disorders and long-term effects on health status during the COVID-19 pandemic and beyond (Martinez-Ferran et al., 2020). In the context of metabolic disorders associated with the COVID-19 pandemic, our findings that patients with hypertensive nephropathy were admitted with increased hemoglobinuria and proteinuria during the COVID-19 pandemic is of great relevance. Previous observational studies have reported an independent association between metabolic syndrome and kidney diseases (Tozawa et al., 2007; Thomas et al., 2011). Therefore, the COVID-19 pandemic should be regarded as a risk factor and additional studies are required to elucidate long-term effects of the COVID-19 pandemic on disorders associated with and contributing to kidney diseases.

The main limitations of our study are the small patient number, no information about intended or postponed admissions from outside primary and secondary care providers and no data on long-term renal survival (increased incidence of ESRD or death). Nevertheless, this is the first report of identifying a subpopulation susceptible to closure of medical services during the COVID-19 pandemic and diagnostic delay of kidney diseases, including hypertensive nephropathy. Therefore, the COVID-19 pandemic should be regarded as a risk factor especially in patients with diseases other than COVID-19 and presenting with constitutional symptoms, primarily admitted to the normal medical ward.

## Data Availability Statement

The original contributions presented in the study are included in the article, further inquiries can be directed to the corresponding author.

## Ethics Statement

The studies involving human participants were reviewed and approved by Ethics committee of the University Medical Center Göttingen (no. 28/9/17). The patients/participants provided their written informed consent to participate in this study.

## Author Contributions

SH and BT conceived the study, collected and analyzed the data, and co-wrote the first draft. SH evaluated histopathological findings. DT collected and analyzed the data. DT and PK participated in the construction and editing of the manuscript. All authors contributed to the article and approved the submitted version.

## Conflict of Interest

The authors declare that the research was conducted in the absence of any commercial or financial relationships that could be construed as a potential conflict of interest.
